# Focal Adhesion of Collagen-Based Bone Grafting Materials Enhances Bone Regeneration

**DOI:** 10.3390/bioengineering12101015

**Published:** 2025-09-24

**Authors:** Mao-Suan Huang, Tzu-Sen Yang, Chia-Jung Wang, John F. Bowley, Wen-Fu T. Lai

**Affiliations:** 1 Department of Dentistry, Taipei Medical University-Shuang Ho Hospital, New Taipei City 235, Taiwan; hms4837@tmu.edu.tw; 2 School of Oral Hygiene, College of Oral Medicine, Taipei Medical University, Taipei City 110, Taiwan; 3 Graduate Institute of Biomedical Optomechatronics, College of Biomedical Engineering, Taipei Medical University, New Taipei City 235, Taiwan; tsyang@tmu.edu.tw (T.-S.Y.); m825111001@tmu.edu.tw (C.-J.W.); 4 International PhD Program in Biomedical Engineering, Taipei Medical University, New Taipei City 235, Taiwan; 5 College of Dentistry, New York University, New York, NY 10010, USA; jfb9473@nyu.edu; 6 Institute of Graduate Clinical Medicine, Taipei Medical University, Taipei City 110, Taiwan; 7 McLean Imaging Center, McLean Hospital and Harvard Medical School, Belmont, MA 02478, USA

**Keywords:** collagen-based materials, cell adhesion, bone tissue engineering

## Abstract

Collagen, which has osteoconductive potential, has been widely used as a scaffold material for bone repair and regeneration for more than the last three decades. Recently, collagen has been combined with other materials to produce collagen-based bone grafting materials with enhanced bone repair and regeneration capacities. However, varied results have been obtained with collagen-based grafting materials. Methods: To elucidate the mechanisms underlying the enhanced bone engineering capacity of these materials, we critically reviewed the current literature on the complex hierarchical structure and properties of native collagen molecules. Results: This review highlights the scientific challenge of manufacturing collagen-based materials with suitable properties and shapes for specific biomedical applications, particularly those related to bone repair and regeneration. Conclusions: This article sheds light on the interactions between collagen and cell receptor molecules to mediate biological pathways. In addition, this article clarifies the mechanisms of cell adhesion-mediated bone regeneration. The findings may guide future research on collagen-based biomaterials.

## 1. Introduction

Except in cases of bone defects caused by an accident, a surgery, or an injury, bone can repair itself by activating surrounding osteoprogenitor cells, leaving no residual scars. However, severe defects that are beyond the bone’s self-healing capacity can lead to bone nonunion, callus formation, and even permanent bone loss. Bone grafts are often used for repairing such defects.

Autologous bone grafting is the gold standard for repairing severe bone defects. Autologous grafts facilitate the repair or regeneration of bone tissue through the differentiation and proliferation of adjacent or transplanted osteoblasts. These grafts are associated with a low risk of rejection, and due to their low antigenicity, the grafts can promote bone regeneration [1]. However, autologous bone grafting has some major disadvantages, such as poor osseointegration with artificial joints or tooth implants. Excessive bone constriction may occur when the defect exceeds 9 cm or when the surrounding tissues fail to provide a sufficient blood supply due to scarring, infection, or irradiation [2]. Moreover, autologous grafting involves the sacrifice of healthy bone tissue at the donor site, resulting in additional morbidity [3]. Harvesting bone from appropriate donor sites requires a complex learning curve and may lead to postoperative complications.

In addition to autologous bone grafts, alternative grafts such as allogenous grafts, xenografts, and allografts are also used for bone repair. These alternative grafts exhibit various physicochemical characteristics and varying reliability in surgical treatments [4,5]. Among all alternative grafts, collagen-based bone grafting materials are widely used in clinical settings. These materials are produced by combining collagen with other materials, thereby increasing bone repair and regeneration capacities.

Bone is a hard connective tissue with a hierarchical structure composed of matrix, cells, and bioactive factors. The bone matrix is mainly composed of type I collagen and hydroxyapatite (HAP). Collagen fibers serve as a template for mineralization and play key roles in determining specific properties, such as compressive and shear behaviors, fracture mode, and toughness, as well as reinforcing bone under external stress from applied loads [6,7].

Osteoblasts are anchorage-dependent cells. Therefore, the initial adhesion of osteoblasts to the biomaterial scaffold is pivotal for their migration, differentiation, proliferation, and subsequent bone formation. The adhesion of cells to the biomaterial surface is a complex process involving cell attachment, cell spreading, and cell–scaffold interaction; this process is called focal adhesion.

Collagen is the most abundant protein, constituting more than one-third (by weight) of the body’s total protein content [8]. Collagen preserves a unique hierarchical structure, constituting a major part of the bone extracellular matrix (ECM) [9]. Type I collagen is the most common type of collagen ECM, particularly in bone tissue [10]. The ECM plays vital roles in the morphogenesis and cellular metabolism of new tissues, conferring mechanical and biochemical properties [11]. The collagen matrix is essential for bone tissue engineering [12] because of its abundance, biocompatibility, high porosity, ability to combine with other materials, easy processing, hydrophilicity, low antigenicity, and absorbability [13,14]. It can promote cell adhesion, differentiation, and proliferation.

Recently, scientists have designed innovative collagen-based biomaterial scaffolds, leveraging the extensive evidence on collagen organization, structure, and properties. The use of these materials has considerably enhanced bone engineering. The design of resorbable collagen-based medical scaffolds requires knowledge of the anatomy and biological function of tissues or organs, as well as an understanding of the role of collagen’s physicochemical properties and structure in tissue or organ regeneration. Different collagen-based scaffolds have been developed for different bone engineering applications. These scaffolds promote biological responses, such as cell signaling, and serve as artificial biomimetic extracellular matrices that guide bone tissue regeneration. The initial step of bone regeneration involves the adhesion of cells to the collagen-based scaffold; the subsequent steps are material–tissue interaction, cell differentiation, cell proliferation, and new bone formation [15,16].

Given the substantial methodological heterogeneity across collagen-based scaffolds—spanning diverse fabrication techniques and inconsistent reporting of key material properties—this review aims to connect collagen–integrin focal-adhesion biology with concrete materials design choices that maximize osteogenesis. To do so, we will identify the most critical collagen–integrin pathways for bone formation, analyze how controllable scaffold variables modulate osteogenic markers, compare the biological and mechanical performance of major fabrication routes, and propose minimal reporting standards to enable robust cross-study synthesis. By clarifying these points, we can better identify optimal, clinically translatable designs. We begin in Section 2 by discussing the foundational mechanisms of cell adhesion, which are activated by the interaction between the collagen matrix and cellular integrins.

## 2. Mechanisms of Collagen Binding and Cell Adhesion

Integrin, a cell surface receptor, plays an essential role in regulating cell signaling, migration, survival, and adhesion to various ECMs, including collagen, fibronectin (FN), and laminin (LN) [17,18,19]. The arginine–glycine–aspartic acid (Arg–Gly–Asp [RGD]), a motif of collagen and fibronectin, constitutes a specific site for its recognition by integrin on cell surface, facilitating cell binding to the ECMs. Integrin, a family of α, β heterodimer, is a key regulator of cell–cell and cell–extracellular microenvironment communication.

Various ECM-binding integrins that facilitate the adhesion of osteoblasts on biomaterial surfaces have been identified. Six integrins play major roles in cell adhesion: α1β1, α2β1, α3β1, αVβ1, α5β1, and α11β1 (Geographic abstract A) [17,20,21,22].

Focal adhesion kinase (FAK) is a cytoplasmic tyrosine kinase that is activated upon integrin binding to the ECM at the site of focal adhesion [23]. Integrin-mediated adhesion at tyrosine 397 leads to the autophosphorylation of FAK, creating a binding site for the proto-oncogene tyrosine protein kinase (Src) homology 2 (SH2) domain of Src; the FAK–Src complex, in turn, phosphorylates other tyrosine residues in FAK, thus maximizing its kinase activity and creating additional protein-binding sites [24]. This active FAK–Src complex activates Ras-related C3 botulinum toxin substrate 1 (Rac1) by recruiting and phosphorylating the p130 Cas scaffold protein (p130 Crk-associated substrate, known as breast cancer anti-estrogen resistance 1 [Bcar1]) [25]. The phosphorylated p130 Cas recruits Dock 180 and engulfment and motility 1 (ELMO1; a *Ced-12* ortholog) by binding to v-crk (sarcoma virus CT10 oncogene homolog crk; proto-oncogene, adaptor protein). The Dock 180-ELMO1 complex functions as an unconventional GEF for Rac1 and promotes the formation of membrane protrusions [26,27]. The activation of Rac1 and cell division cycle 42 (Cdc42) is also inhibited. This suppresses actomyosin contractility and enhances actin-mediated protrusion. The activity of Rac1 and Cdc decreases, whereas that of RhoA gradually increases, promoting the formation of stress fibers and the maturation of focal adhesion (Geographic abstract C) [28,29].

The FAK–Src complex also phosphorylates paxillin kinase linker (the GEF for Cdc and Rac1, which is also known as G-protein-coupled receptor kinase-interacting protein 2 [GIT2], Pak-interacting exchange factor-beta [βPIX, also known as cloned out of library-1 [30], and Rho guanine nucleotide exchange factor 7 {Gef7}]). βPIX recruits and activates Rac1 through direct interactions with focal adhesions and membrane protrusions [31]. Notably, paxillin kinase linker and βPIX are phosphorylated by Src, which further modulates their activity in response to integrin-mediated adhesion [30,32]. Thus, integrin signaling through Src family kinases (SFKs) can regulate the localization and activity of GEFs, thereby controlling the formation of membrane protrusions.

The FAK–Src complex activates several pathways; subsequently, activated Rac and Cdc42 GTPases facilitate the formation of a membrane protrusion at the site of integrin binding in the early stages of cell spreading. Simultaneously, this complex, together with syndecans, mediates the suppression of actomyosin contractility by reducing the activity of RhoA. In the later stages of cell spreading, integrins promote the activity of several GEFs, leading to a shift in the balance between RhoA and Rac1 activity in favor of RhoA activity, thereby enhancing RhoA-mediated actomyosin contractility. The integrin α5β1 is particularly efficient in promoting the second phase of cell spreading, which may involve the force-induced activation of SFKs.

The enhanced activity of FAK promotes RhoA activity, thereby promoting cellular contractility through ROCK. Crosstalk between RhoA–ROCK and the extracellular signal-regulated kinase (ERK)–mitogen-activated protein kinase (MAPK) pathway induces the phosphorylation of p44/42 MAPK (ERK1/ERK2) through MAPK kinase (MEK), which regulates the activity of the osteogenic transcription factor runt-related transcription factor 2 (RUNX2). RUNX2 controls the expression of osteogenic genes such as osteocalcin (OCN), alkaline phosphatase (ALP), and bone sialoprotein, ultimately driving differentiation toward mature osteoblasts (Geographic abstract D) [33].

## 3. Collagen-Based Materials

Collagen-based materials promote the differentiation of human-induced pluripotent stem cells into osteoblasts [34]. The collagen matrix, combined with other ECM or materials, also enhances cell differentiation and proliferation. Other ECMs or materials include LN [35], FN [36,37], chitosan (CS) [38], HAP [39,40,41], calcium phosphate cement (TCP), hydrogel [42], fibrin, and fibrinogen [43]. Collagen-based materials are also applied as a coating on implants to enhance osteoblast adhesion to the implant surface and osseointegration between bone and the implant [44,45,46,47,48,49]. The techniques and mechanisms are described in the following sections and Table 1.

### 3.1. Orientation of Collagen Fibers

The orientation of collagen-coated poly-lactide-co-glycolide (PLGA)/poly-caprolactone (PCL) fibers was reported to enhance bone regeneration through cell adhesion. Chen et al. demonstrated that adipose-derived stem cells exhibited the highest expression of adhesion-related genes, such as those encoding integrin β1, cadherin 11, Fn-1, LN, and N-cadherin [50]. Electrospinning technology was used to fabricate collagen/HAP fibers to improve bone regeneration [51]. Collagen/HAP fibers support cell adhesion and bone regeneration. Yuanyuan Zhou et al. produced collagen/HAP composite fibers through electrospinning, and after 6 days of culture, the composite fibers exhibited higher viability and ALP activity than myeloma cells [52].

### 3.2. LN

Selective cell retention has been widely used as a bone tissue engineering technique. LN is a main component of the ECM and the basement membrane. LN plays a key role in mediating cell–matrix adhesion, leading to cell proliferation and differentiation [34,35]. A collagen-binding domain (CBD) containing the core functional amino acid sequences of LN-4 (CBD-LN peptide) was introduced on the functional surface of a collagen-based decalcified bone matrix scaffold. The decalcified bone matrix/CBD-LN scaffold maintained osteoblast proliferation and induced osteogenic differentiation through early cell adhesion mediated by upregulated integrin β1 expression [35]. In tissue engineering, bioactive molecules have been introduced into three-dimensional porous scaffolds to mimic the in vivo microenvironment.

### 3.3. FN

FN is an ECM glycoprotein with a size ranging from 230 to 270 kDa. The dimer is formed by α and β subunits. FN includes types I, II, and III domains. The type III domain is the most sensitive to unfolding. Several receptors can bind to FN—for example, α2β1, α3β1, αvβ1, and α5β1—and improve focal adhesion, cell proliferation, and cell migration [53]. FN combined with type I collagen can promote focal adhesion, as evidenced by elevated filopodium formation, increased cell circularity, and accelerated spreading in the mesenchymal stromal cell line [36]. Coating FN combined with OCN on the collagen matrices enhanced the adhesion of osteoblast-like cells (MC3T3-E1) and the mRNA levels of osteogenic markers, such as RUNX2, ALP, and collagen type I, in these cells [37].

### 3.4. Ceramic and Combined Materials

In a previous study, a hybrid scaffold composed of granular HAP and collagen was designed to mimic the microenvironment for the adhesion, viability, and osteoinduction of human bone marrow-derived mesenchymal stem cells (BMSCs) [54,55].

Moreau et al. reported that collagen incorporated into calcium phosphate bone cement increased the attachment and osteogenesis of osteoblasts [56]. Gutierrez et al. [57] proved the adhesion activity of HAP. The nano apatite–collagen composite appeared more similar to natural bone in terms of biomimetics than nano apatite cement without collagen [44,56,58]. Functionally graded CO_3_ apatite–collagen containing magnesium (FGMg-Ap-collagen) was also reported to enhance osteoblast adhesion to apatite and to promote bone formation [59]. CS combined with collagen/HAP in mesenchymal stem cells (MSCs) [60] and rats [61], and collagen/β-TCP in the human osteoblast cell line MG63 [62], increased cell adhesion and proliferation, resulting in osteogenesis.

Yu et al. reported that intrafibrillar mineralized Col-HA-based scaffolds, constructed with either cellular or lamellar microstructures, exhibited enhanced bone regeneration capacity in a mouse model. Moreover, Fe/Mn incorporation promoted the osteogenic potential of the lamellar scaffolds, facilitating the in vitro osteogenic differentiation of BMSCs and the in vivo bone regeneration in the presence of fresh bone marrow cells [63].

### 3.5. CS

CS, a nontoxic natural polymer, is primarily composed of 1,4-linked *N*-acetyl-D-glucosamine and D-glucosamine units. CS exhibits high solubility in dilute acidic solutions with a pH < 6.5 [64]. This polymer forms covalent bonds with FN, improving cell adhesion [65]. CS binding to collagen/HAP or CS/collagen/β-TCP leads to the formation of a three-dimensional structure enhancing cell adhesion and proliferation, then resulting in osteogenesis [61,62]. Osteoblast adhesion increases with increasing β-TCP and CS contents [62,66].

### 3.6. Fibrin and Fibrinogen

Fibrin is widely used for enhancing the focal adhesion of collagen [67]. Santos et al. reported that the combination of collagen sponge and fibrin glue exerted hemostatic effects and ensured more favorable bone formation than collagen sponge only [67,68]. B.-S. Kim et al. found that type I collagen-derived atelocollagen/fibrin composite gel, combined with an optimal concentration of fibrinogen, supported human MSC growth in vitro and bone formation in vivo [69].

### 3.7. Cytokines and Chemokines

Cytokines or chemokines, such as bone morphogenetic proteins (BMPs), chemotactic cytokine ligand (CXCL)12, and CXCL13, can enhance cell adhesion to the scaffold. Osteoblast adhesion to collagen or collagen-based materials leads to increased cell proliferation. In a previous study, BMP4 was immobilized in a CBD and bound to the collagen–polyglycolic hybrid scaffold; the BMP-immobilized hybrid scaffold supported the adhesion and proliferation of MSCs [70].

Claude Laflamme et al. revealed that a mixture of BMP-2/BMP-7 homodimers enhanced osteoblast adhesion and growth following culture on a collagen scaffold. Osteoblast adhesion and proliferation increased 4 days after culture on the collagen scaffold. BMP-2/BMP-7 promoted bone regeneration through different mechanisms involving interleukin-6 and matrix metalloproteinase inhibitors [71].

Sylvia Weeks et al. reported that the incorporation of the chemokines CXCL12 and CXCL13 into a poly-L-lactic acid–collagen-based scaffold increased cell adhesion. Combining CXCL12 with an FN- and collagen-coated scaffold increased MSC adhesion to the scaffold through α4 and α5β1 binding [72]. This scaffold promoted the differentiation of MSCs into osteoblasts, resulting in bone formation.

### 3.8. Small Molecules

Grafting collagen with other materials to fabricate a scaffold is an effective strategy for enhancing cell proliferation and ALP expression [73]. Collagen can improve cell adhesion because of the presence of its Pro-α3(V) chain in bone. Through the N-terminal peptide of this chain, collagen adheres to osteosarcoma cells [74]. OCN, a small bone ECM protein, accelerated bone formation in a rat model when added to HAP/collagen composites. Histological findings indicate that OCN activates both osteoblasts and osteoclasts during early bone formation [39].

### 3.9. Implants

Collagen is widely used to coat biomedical implants. Geissler and Hempel et al. reported that the collagen type I coating of Ti6Al4V promoted the initial adhesion of osteoblasts in the presence of fetal calf serum. Moreover, 60% to 90% of all osteoblasts adhered to collagen type I-coated surfaces, whereas 30% of the initial cell number remained adhered to uncoated surfaces. Collagen type I, which includes the RGD peptide, effectively promotes cell adhesion by interacting with α1β1 and α2β1 [45]. The type I collagen-coated Ti–6Al–4V alloy facilitated osseointegration and bone-to-implant contact [46].

Titanium-based implants exhibit osseointegration and are widely used in dental and orthopedic care. However, the cell growth and differentiation capacity on the surface of titanium-based implants are limited. To address these limitations, various functionalization strategies for titanium surfaces have been developed, for example, bioactivated coatings. One of the most used peptides for functionalizing biomaterials is a cell adhesion peptide containing the RGD sequence, which is found in collagen, FN, and bone sialoprotein. RGD peptide-enriched materials interact with integrin on the cell surface and can enhance the proliferation of osteoblasts and BMCs (bone marrow-derived stem cells) [47].

In dental care, zirconia is used to manufacture implants. To enhance implant compatibility, nano-HAP is first bound to the surface, and then type I collagen is immobilized on the surface. Compared with cases with no coating or with nano-HAP-only coating, cases with nano-HAP–collagen coating exhibited increased osteoblast attachment and spreading on the surface; higher osteoblast differentiation was confirmed by higher ALP activity and mineralization [48]. HAP and extensive collagen coating of an implant surface can facilitate cell attachment due to the presence of increased hydroxyl groups on the surface, which results in the formation of a low contact angle and the activation of carboxylic groups, which are beneficial for osteoblast adhesion and proliferation [49].

Across the reviewed studies, methodological heterogeneity is substantial: collagen scaffolds are fabricated via diverse routes (e.g., electrospinning, surface functionalization/coatings, and hybrid composites), and key design variables (collagen fraction, crosslinker chemistry, fiber alignment/porosity, and mineral content) are reported with inconsistent parameters. Nevertheless, a consistent signal emerges across platforms: collagen-based constructs enhance osteoblast adhesion and spreading, elevate early osteogenic readouts (e.g., ALP), and support bone formation.

Beyond preclinical and experimental studies, collagen-based bone grafting materials have already been translated into diverse clinical settings. In dental and maxillofacial surgery, collagen-containing composites like Bio-Oss^®^ Collagen are widely used for socket preservation, ridge augmentation, and sinus floor elevation, demonstrating predictable bone regeneration and enhanced handling properties [75]. In orthopedics, collagen scaffolds—in combination with osteoconductive ceramics such as hydroxyapatite or β-tricalcium phosphate—support bone healing in non-union fractures and spinal fusion, leveraging their favorable biocompatibility and graft integration [76]. Recent advances further highlight the potential of collagen scaffolds engineered with novel functionalities, such as piezoelectric properties to promote osteoinduction [77], and their integration with 3D printing technologies to improve biomechanical performance and clinical applicability [78]. Moreover, the exploration of alternative sources, including marine-derived collagen, offers new opportunities for sustainable and safe biomaterial development in bone tissue repair [79]. Despite these advances, challenges remain, including achieving optimal degradation rates, maximizing mechanical strength, and enabling tailored, patient-specific scaffold designs to improve long-term outcomes.

Despite extensive progress in the development of collagen-based scaffolds for bone regeneration, several limitations remain to be addressed before their widespread clinical translation. Collagen-based scaffolds have shown remarkable promise in bone regeneration due to their biocompatibility, biodegradability, and ability to mimic the native extracellular matrix. However, their clinical translation still faces notable limitations, including insufficient mechanical strength for load-bearing applications, variability in degradation rates, and batch-to-batch inconsistency arising from natural sources. In addition, collagen alone provides limited osteoinductive capacity, often necessitating combination with bioactive ceramics, growth factors, or stem cells to achieve robust bone regeneration. Looking forward, the prospects for collagen-based scaffolds lie in advanced fabrication strategies—such as 3D bioprinting, nanostructure modification, and incorporation of signaling molecules—that can precisely tailor scaffold architecture and biological performance. Integration with smart biomaterials and controlled drug delivery systems further offers exciting opportunities to enhance osteogenic outcomes and support patient-specific, precision therapies in regenerative medicine.

**Table 1:** Studies on collagen-based grafting materials.

Characterization	Technology	Results	Article Title	Authors	Publisher
**Orientation** Alignment of collagen-based scaffold Bone-mimetic-oriented (type I) collagen scaffolds	Using extrusion to obtain collagen and then fabricating the scaffold	Human induced pluripotent stem cell-derived osteoblasts exhibited favorable responses to the collagen scaffolds, as confirmed by the actin structure	Superior alignment of human iPSC-osteoblasts associated with focal adhesion formation stimulated by oriented collagen scaffold	Ryosuke Ozasa et al. [34]	International Journal of Molecular Sciences (June 2021)
**Orientation** Collagen-based scaffold and PLGA, PCL through electrospinning PLGA/PCL/type I collagen electrospun scaffolds	The electrospun scaffold made of polymer contained type I collagen	Upregulated expression of adhesion-related genes (β1, Cadherin 11, and Fn-1), with ADSC adhesion	Enhanced osteogenesis of ADSCs by the synergistic effect of aligned fibers containing collagen I	Chen et al. [50]	ACS Applied Materials & Interferences (October 2016)
**Orientation** Collagen-based scaffold and HAP through electrospinning Electrospinning of collagen/HAP fibrous composite	HAP mixed with type I collagen	Cells exhibited increased viability on the collagen/HAP composite nanofibers	Greener synthesis of electrospun collagen/ hydroxyapatite composite fibers with an excellent microstructure for bone tissue engineering	Yuanyuan Zhou et al. [52]	International Journal of Nanomedicine (April 2015)
**Orientation** Poly(lactide-co-glycolide)/CS scaffolds with collagen	Immersed scaffold in a solution containing type I collagen	Cell adhesion efficiency increased by approximately 1.2-fold; promotion of stem cell differentiation into osteoblasts	Effect of surface-modified collagen on the adhesion, biocompatibility and differentiation of bone marrow stromal cells in poly(lactide-co-glycolide)/CS scaffolds	Yung-Chih Kuo et al. [14]	Colloids and Surfaces B: Biointerfaces (October 2010)
**Laminin** Collagen-based scaffold and laminin Collagen-based decalcified bone matrix scaffold modified with laminin α4	Collagen-binding domain (CBD) containing laminin alpha 4 on the scaffold	Promotion of early cell adhesion	Laminin alpha 4 promotes bone regeneration by facilitating cell adhesion and vascularization	Yong Tang et al. [35]	Acta Biomaterialia (March 2021)
**Fibronectin** Fibrillar complexes based on collagen type I and fibronectin	Fibronectin solution was added to the collagen solution; then, KH_2_PO_4_ was added to form fibril shapes	MSCWJ-1 cells were elongated and had increased area on the composite fibril, which was confirmed by the actin cytoskeleton	The structural interactions of molecular and fibrillar collagen type I with fibronectin and its role in the regulation of mesenchymal stem cell morphology and functional activity	Yuliya Nashchekina et al. [36]	International Journal of Molecular Sciences (October 2022)
**Fibronectin** Fusion protein, human OCN (hOCN) with FN_III9–10_ combines with collagen	rhOCN/FN_III9–10_ was crosslinked with collagen to form the matrix	rhOCN/FN_III9–10_-functionalized collagen matrix increased not only the adhesion but also the differentiation of MC3T3-E1 cells	Osteocalcin/fibronectin-functionalized collagen matrices for bone tissue engineering	Kim S. et al. [37]	Journal of Biomedical Materials Research Part A, (October 2015)
**Ceramic and combined materials** Collagen-based scaffold with silicon and HAP Silicon, collagen, and HAP	Silicon, collagen, and HAP	After 7 days, osteoblasts exhibited similar interaction with the scaffold and bovine bone	Analysis of in vitro osteoblast culture on scaffolds for future bone regeneration purposes in dentistry	Sandra J. Gutie’rrez-Prieto et al. [57]	Advances in Pharmacological Sciences (2019)
**Ceramic and combined materials** Collagen-based scaffold and a mixture of tetracalcium phosphate and dicalcium phosphate anhydrous Calcium phosphate bone cement (CPC) with type I bovine collagen	CPC powder mixed with collagen powder	Two-fold increase in osteoblast attachment	Self-setting collagen-calcium phosphate bone cement: Mechanical and cellular properties	Jennifer L. Moreau et al. [56]	Journal of Biomedical Materials Research Part A (July 2008)
**Ceramic and combined materials** Collagen-based scaffold and FGMgCO_3_Ap FGMgCO_3_Ap and atelocollagen composite pellet	FGMgCO_3_Ap mixed with atelocollagen	Osteoblast-like cells adhered more effectively to the composite than to the Ti plate	Action of GMgCO_3_Ap-collagen composite in promoting bone formation	Y. Yamasaki et al. [59]	Biomaterials (May 2023)
**Ceramic and combined materials** Collagen-based scaffold with CS and HAP Collagen/CS sponges (composed of collagen, CS, and HAP)	Homogenization of the collagen gel, CS gel, and HAP	Collagen coating and RGD coating exhibited good compatibility	Use of collagen/CS sponges mineralized with hydroxyapatite for the repair of cranial defects in rats	M.A.S. Munhoz et al. [61]	Injury (September 2018)
**Ceramic and combined materials** Collagen-based scaffold with HA Collagen–HAP scaffold combined with Fe^2+^ or Mn^2+^ ions	The scaffolds made of poly(acrylic acid) and type I collagen and then substituted with Fe^2+^ or Mn^2+^ were shaped as a disc piece whose diameter and thickness were 5.5 and 1 mm, respectively	MC3T3 cells exhibited viability and attachment when collagen was used; the parameters improved when Mn^2+^ and Fe^2+^ were added, as confirmed by the formation of pseudopodia	Intrafibrillar mineralized collagen-hydroxyapatite-based scaffolds for bone regeneration	Le Yu et al. [63]	ACS Applied Material & Interfaces (December 2020)
**Ceramic and combined materials** Collagen-based scaffold with HAP Collagen–hemostat and granular HAP scaffold	The scaffold was prepared by mixing granular HAP and collagen hemostat and then dried overnight	After 21 days, human bone marrow-derived mesenchymal stem cells exhibited higher growth on the scaffold and exhibited high viability and cytoskeleton structure as the cell attachment	Enhanced osteogenic differentiation of human bone marrow-derived mesenchymal stem cells by a hybrid HAP/collagen scaffold	Elisa Mazzoni et al. [55]	Frontiers in Cell and Develop Bio (January 2021)
**CS** Collagen-based scaffold with β-TCP and CS Collagen, β-tricalcium phosphate, and CS matrix	Different ratios of CS and β-TCP formed with collagen	Composite made of β-TCP/collagen led to enhanced cell adhesion and mechanical properties	Bioactivity and mechanical properties of collagen composite membranes reinforced by CS and β-TCP	Sang-Bae Lee et al. [62]	Society For Biomaterials (April 2012)
**Fibrin and fibrinogen** Collagen-based fibrin Fibrin–collagen sponges	A fibrin–collagen sponge was immersed in fibronectin–gelatin solution to generate fibrin	Favorable cell attachment and increased ALP activity	Improvements of osteoblast adhesion, proliferation, and differentiation in vitro via fibrin network formation in collagen sponge scaffold	Beom-Su Kim et al. [67]	J Biomedical Materials Research Part A (July 2013)
**Fibrin and fibrinogen** Collagen-based scaffold with fibrin glue–modified collagen sponge	Fibrin glue composed of human fibrinogen, aprotinin, and thrombin	The sponge promoted new bone formation in a rat model of calvarial bone defect	Effect of collagen sponge and fibrin glue on bone repair	Thiago de Santana SANTOS et al. [68]	J Appl Oral Sci. (September 2015)
**Cytokine and chemokine** Collagen-based scaffold and PLGA with BMP-4 Bone morphogenetic protein-4 immobilized in a collagen–PLGA hybrid scaffold	PLGA was crosslinked to type I collagen and then immersed in BMP-4	Mesenchymal stem cells adhered to the scaffold and exhibited uniform distribution on the scaffold with BMP-4	Spatial immobilization of bone morphogenetic protein-4 in a collagen-PLGA hybrid scaffold for enhanced osteoinductivity	Hongxu Lu et al. [70]	Biomaterials (June 2012)
**Cytokine and chemokine** Collagen-based scaffold with BMP-2 and BMP-7 CollaTape scaffolds	CollaTape (taken from bovine deep flexor [Achilles] tendon)	The collagen scaffold with BMP-2/BMP-7 promoted osteoblast adhesion	Effect of BMP-2 and BMP-7 homodimers and a mixture of BMP-2/BMP-7 homodimers on osteoblast adhesion and growth following culture on a collagen scaffold	Claude Laflamme et al. [71]	Biomedical Materials (February 2008)
**Cytokine and chemokine** Collagen-based scaffold with PLLA and chemokines Type IV collagen and some chemokines were coated on the scaffold made of PLLA	PLLA-coated coverslips were incubated with fibronectin, type IV collagen, or heparin with chemokines (CXCL12 and CXCL13)	Combined CXCL12 and collagen enhanced cell adhesion compared with the outcomes noted with collagen alone	The effects of chemokine, adhesion and extracellular matrix molecules on binding of mesenchymal stromal cells to poly(L-lactic acid)	SYLVIA WEEKS et al. [72]	Cytotherapy (May 2012)
**Small molecule** Collagen-based scaffold and HAP with osteocalcin HAP/collagen composites help in the secretion of osteocalcin in the scaffold or electrospinning	Nanocrystalline HAP implants contained 2.5% type I collagen/graphene oxide, HAP combined with collagen	The expression of adhesion proteins (osteopontin, bone sialoprotein, and CD44) increased; electrospinning-coated alloys increased cell adhesion and viability	Osteocalcin enhances bone remodeling around hydroxyapatite/collagen composites; novel hydroxyapatite/graphene oxide/collagen bioactive composite coating on Ti16Nb alloys by electrodeposition	Stefan Rammelt et al. and Yılmaz, E. et al. [39,40]	Journal of Biomedical Materials Research Part A (March 2005); Materials Science and Engineering: C (2019)
**Small molecule** Collagen-based scaffold and PLGA, HAP with BMP-4 Nano-HAP–poly(D,L-lactide-co-glycolide)–collagen biomaterial	Multistep polymerization and fabrication process	Increased cell proliferation and ALP expression	Mechanical properties and osteogenic potential of Hydroxyapatite-PLGA-collagen biomaterial for bone regeneration	Didarul B. Bhuiyan et al. [73]	Journal of Biomaterials Science (May 2016)
**Implant** Collagen was coated on the alloy Collagen type I coating of Ti6Al4V	Ti6Al4V alloy coated with type I collagen	The alloy coated with type I collagen enabled osteoblasts to attach better and faster; they were recognized by integrins α1β1 and α2β1	Collagen type I coating of Ti6Al4V promotes adhesion of osteoblasts	Geißler et al. [45]	J Biomed Mater Res (2000).
**Implant** Collagen was coated on the alloy Ti–6Al–4V alloy combined with collagen	Ti–6Al–4V alloy coated with type I collagen	The alloy coated with type I collagen led to high levels of new bone formation	An alternative ex vivo method to evaluate the osseointegration of Ti–6Al–4V alloy also combined with collagen	Francesca Veronesi et al. [46]	Biomedical Materials (February 2021)
**Implant** Collagen crosslinked to alloy Collagen crosslink on titanium (Ti6Al4V) surfaces	Different crosslinkers (EDC/NHS, riboflavin, and lysyl oxidase) were used for coupling the collagen with the alloy	Cells exhibited favorable attachment to the material surface modified by crosslinkers, which was confirmed through immunofluorescence	Transglutaminase enables highly hydrolytically and proteolytically stable crosslinking of collagen on titanium surfaces and promotes osteogenic differentiation of human mesenchymal stem cells	Alena L. Palkowitz et al. [47]	Society For Biomaterials (December 2023)
**Implant** Collagen was immobilized on the alloy Zirconia dental implants coated with collagen	Coating the surface with nano-HAP and then immobilizing type I collagen on it	Compared with cases with no coating and with nano-HAP-only coating, those with nano-HAP–collagen coating exhibited increased osteoblast attachment, spreading, mineralization, and differentiation	Bioactive surface of zirconia implant prepared by nano-hydroxyapatite and type I collagen	Hun Kim et al. [48]	Coatings (September 2022)
**Implant** Collagen was immobilized on alloy Stainless steel (SS316L) immobilized with HAP, collagen on stainless steel (SS316L) with polydopamine	HAP and collagen immobilized on polydopamine and then grafted on the implant surface	The presence of hydroxyl groups on the surface, resulting in a low contact angle and carboxylic group activation, may be beneficial for osteoblast adhesion and proliferation	Synthesis and characterization of collagen–hydroxyapatite immobilized on polydopamine grafted stainless steel	Zafirah Tapsir et al. [49]	Surface and Coatings Technology (January 2016)

Abbreviations: CS, chitosan; HAP, hydroxyapatite.

## 4. Conclusions

Over the last decades, extensive research has been conducted on collagen-based materials to improve their biological and mechanical properties for supporting efficient bone regeneration. Current advanced strategies are investigated in this review to figure out how to modify properties of materials in order to achieve a significant bone engineering.

This review integrates molecular cell adhesion mechanisms with materials science technology to advance the design of collagen-based scaffold for bone repair and regeneration, emphasizing that focal adhesion initiated by specific collagen–integrin interactions (e.g., α1β1, α2β1, α11β1) triggers downstream FAK–MAPK–RUNX2 signaling cascades, which regulate osteoblast differentiation and the expression of osteogenic genes such as ALP, osteocalcin, and bone sialoprotein. By linking these mechanistic pathways to scaffold design parameters, this work provides a unified framework for understanding and optimizing collagen-based biomaterials, with specific strategies including tailoring fiber orientation and surface chemistry to promote integrin-specific adhesion, hybridizing collagen with bioactive ceramics (e.g., HAP, β-TCP, FGMg-Ap) and polymers (e.g., chitosan, PLGA) to enhance mechanical stability and biological signaling, incorporating bioactive molecules such as BMPs, chemokines, and RGD-containing peptides to accelerate osteogenesis, and functionalizing implant surfaces with collagen-based coatings to improve osseointegration.

Despite substantial advances, key research gaps remain, including the need for high-throughput screening platforms to evaluate integrin-specific scaffold responses, long-term in vivo validation of collagen–integrin-targeted designs, and scalable manufacturing processes that preserve collagen’s native hierarchical structure; addressing these challenges will accelerate the translation of next-generation collagen-based scaffolds into clinical practice. Overall, this review provides both a mechanistic synthesis and a set of actionable guidelines for scaffold design, offering value to researchers in biomaterials, tissue engineering, and regenerative medicine and underscoring the promise of integrating molecular pathways with engineering principles to develop biomimetic scaffolds capable of reliably guiding bone regeneration.

## Data Availability

No new data were generated or analyzed in the article.
